# Identification of Wheat Yellow Rust Using Optimal Three-Band Spectral Indices in Different Growth Stages

**DOI:** 10.3390/s19010035

**Published:** 2018-12-21

**Authors:** Qiong Zheng, Wenjiang Huang, Ximin Cui, Yingying Dong, Yue Shi, Huiqin Ma, Linyi Liu

**Affiliations:** 1 College of Geosciences and Surveying Engineering, China University of Mining and Technology (Beijing), Beijing 100083, China; zhengqiong@student.cumtb.edu.cn; 2 Key Laboratory of Digital Earth Science, Institute of Remote Sensing and Digital Earth, Chinese Academy of Sciences, Beijing 100094, China; dongyy@radi.ac.cn (Y.D.), shiyue@radi.ac.cn (Y.S.); mahq0712@nuist.edu.cn (H.M.); liuly35@radi.ac.cn (L.L.); 3 State Key Laboratory of Remote Sensing Science, Institute of Remote Sensing and Digital Earth, Chinese Academy of Sciences, Beijing 100094, China; 4 University of Chinese Academy of Sciences, Beijing 100049, China; 5 Collaborative Innovation Center on Forecast and Evaluation of Meteorological Disasters, Nanjing University of Information Science & Technology, Nanjing 210044, China

**Keywords:** yellow rust disease, different growth stages, three-band spectral index, wheat infection, hyperspectral remote sensing

## Abstract

Yellow rust, a widely known destructive wheat disease, affects wheat quality and causes large economic losses in wheat production. Hyperspectral remote sensing has shown potential for the detection of plant disease. This study aimed to analyze the spectral reflectance of the wheat canopy in the range of 350–1000 nm and to develop optimal spectral indices to detect yellow rust disease in wheat at different growth stages. The sensitive wavebands of healthy and infected wheat were located in the range 460–720 nm in the early-mid growth stage (from booting to anthesis), and in the ranges 568–709 nm and 725–1000 nm in the mid-late growth stage (from filling to milky ripeness), respectively. All possible three-band combinations over these sensitive wavebands were calculated as the forms of PRI (Photochemical Reflectance Index) and ARI (Anthocyanin Reflectance Index) at different growth stages and assessed to determine whether they could be used for estimating the severity of yellow rust disease. The optimal spectral index for estimating wheat infected by yellow rust disease was PRI (570, 525, 705) during the early-mid growth stage with R^2^ of 0.669, and ARI (860, 790, 750) during the mid-late growth stage with R^2^ of 0.888. Comparison of the proposed spectral indices with previously reported vegetation indices were able to satisfactorily discriminate wheat yellow rust. The classification accuracy for PRI (570, 525, 705) was 80.6% and the kappa coefficient was 0.61 in early-mid growth stage, and the classification accuracy for ARI (860, 790, 750) was 91.9% and the kappa coefficient was 0.75 in mid-late growth stage. The classification accuracy of the two indices reached 84.1% and 93.2% in the early-mid and mid-late growth stages in the validated dataset, respectively. We conclude that the three-band spectral indices PRI (570, 525, 705) and ARI (860, 790, 750) are optimal for monitoring yellow rust infection in these two growth stages, respectively. Our method is expected to provide a technical basis for wheat disease detection and prevention in the early-mid growth stage, and the estimation of yield losses in the mid-late growth stage.

## 1. Introduction

Yellow rust disease, caused by the fungus *Puccinia striiformis*, is a serious threat to wheat production and impacts the yield and quality of wheat [1,2]. The disease is known to occur in more than 60 countries worldwide and is the most important wheat disease in China [3]. In extreme situations of very susceptible cultivars and under favorable weather conditions, yellow rust can reduce the yield by 100% [4]. Conventional stress-detection methods usually range from detection by the naked eye to random monitoring, which is highly subjective, labor intensive, and time consuming. Even worse, when management and policy decisions are based on imprecise and inaccurate data from traditional monitoring results to evaluate the damage, it may cause costly mistakes [5]. In precision agriculture, the timely detection of crop diseases at different growth stages are critical to the effective management of the economy and agriculture [6].

Advancements of remote-sensing techniques provide opportunities for the non-destructive detection of plant diseases, especially hyperspectral technology [7]. Hyperspectral analysis is an ideal tool to capture biophysical variations caused by infestations of crops on account of its abundant narrow bands and high spectral resolution. These advantageous characteristics have been proven to enable information about plant growth to be obtained efficiently and to discriminate among diseases [8,9,10]. Hyperspectral remote sensing can detect subtle changes in the biophysical and biochemical characteristics of plants caused by various types of stress [11]. For example, Das et al. [12] pointed out that the four wavebands of 760 nm, 990 nm, 680 nm, and 540 nm could be used to significantly distinguish rice infected with bacterial leaf bight disease from healthy rice by using stepwise discrimination analysis. Mahlein et al. [11] proposed that the sugar beet rust index composed of spectral information at 570 nm, 513 nm, and 704 nm based on the RELIEF-F algorithm could distinguish sugar beet rust from other diseases. In previous studies concerned with yellow rust disease, Moshou et al. [1] found that the wavebands centered at 680 nm, 725 nm and 750 nm were the most sensitive bands for yellow rust detection. Bravo et al. [13] used a quadratic discriminating model combined with the sensitive wavebands (at 543 ± 10 nm, 630 ± 10 nm, 750 ± 10 nm, and 861 ± 10 nm) for yellow rust discrimination with the coefficient of determination of 0.96. Although the spectral reflectance can reflect the physiological status of plants for disease detection based on in situ hyperspectral analysis, the spectral data also contain information about the canopy structure and soil background. 

Vegetation indices that combine the sensitive bands in a certain mathematical form can enhance the reflectance sensitivity of plant parameters and reduce the effects of various types of background interference. These indices can be used to estimate crop yield [14,15], detect variations in the leaf area index [16,17], biophysical variables [18,19], and identify crop diseases [20,21]. Several spectral indices drawn from the literature have shown potential for plant disease detection. For instance, Huang et al. [22] reported that the spectral vegetation index, the photochemical reflectance index (PRI), was strongly correlated with the yellow rust disease index in wheat. Devadas et al. [4] found that the anthocyanin reflectance index (ARI) could distinguish wheat infected by yellow rust from healthy wheat and that affected by other rust diseases (leaf rust and stem rust). Rumpf et al. [23] detected beet diseases in the earliest stages based on a support vector machine (SVM) and a spectral index. Several vegetation indices can be used to detect yellow rust in wheat. These results indicated that spectral reflectance and vegetation indices can be used as a non-destructive remote-sensing technique to detect different crop pests and diseases. However, the spectral indices they proposed may be only suitable for several growth stages or even for one stage of the growth period of wheat. 

In practice, the characteristics of wheat at different growth stages of post-infection yellow rust are different. The first symptoms of yellow rust disease are the appearance of yellow spots on the upper side of wheat leaves. With on-going pathogenesis these spots become yellow and bacteria spores are formed. These symptoms begin by manifesting themselves in yellow, orange, and then dark brown colors. The final symptom is dry leaf [4,24]. The disease reduces plant vigor and may cause it to become withered or die [25]. The canopy growth status changes during the growth period, and may result in an inconsistent relationship between the vegetation index and the status of yellow rust at different growth stages. At present, many researchers assumed the crop population to be a homogeneous body during the entire growth period; thus, they usually explored the sensitive bands and constructed a vegetation index for disease discrimination by pooling the observed data from different growth stages for the entire growth period [26]. The study of yellow rust in wheat during the entire growth period is attracting less attention. Therefore, finding the optimal vegetation index may contribute to enhancing the accuracy and stability of monitoring models for wheat affected by yellow rust at different growth stages. 

Our study aimed to detect yellow rust disease in wheat during different growth stages. The objectives of this study were to: (1) illustrate the response characteristics of hyperspectral reflectance under yellow rust infection at different growth stages over the spectral range 350–1000 nm; (2) discuss the sensitivity of spectral indices to discriminate wheat affected by yellow rust at different growth stages; (3) develop optimal three-band spectral indices for yellow rust discrimination during different growth stages; (4) evaluate the performance of these new indices. Monitoring yellow rust in wheat is helpful for guiding crop production, which would facilitate effective precision agricultural research and management.

## 2. Materials and Methods

### 2.1. Experimental Area

Experiment 1 (Exp. 1): The canopy experiments were conducted at Beijing Xiaotangshan Precision Agriculture Experiment Base, in Changping District, Beijing (40°10.6′N, 116°26.3′E) during the 2002–2003 growing seasons. The soil had an approximate nutrient content of 1.42–1.48% of organic matter, 0.08–0.10% of total nitrogen, 58.6–68.0 mg/kg of alkali-hydrolysis nitrogen, 20.1–55.4 mg/kg of available phosphorus, and 117.6–129.1 mg/kg of rapidly available potassium. The wheat cultivars “Jing 411”, “98-100”, and “Xuezao” were selected for their varied resistance to yellow rust, i.e., “Jing 411” has strong resistance to yellow rust, “98-100” has moderate resistance, and “Xuezao” is highly susceptible to infection. Seeds were sown in rows on 4 September 2002. The wheat was inoculated with yellow rust pathogen (3, 9, 12 mg/100 mL spore solution) on 4 April using spores inoculated to induce different severity of yellow rust disease according to the National Plant Protection Standards. The wheat area of each different inoculation concentration is about 1.2 ha with an even constitution of the three cultivars. In the yellow rust experiment, the distance between control and inoculation plots was approximately 5 m, with the control plots treated with pesticides to prevent infection. In this study, the different plots were initiated in the same way with 200 kg/ha nitrogen and 450 m^3^/ha water and thereafter managed under the same conditions.

Experiment 2 (Exp. 2): A series of canopy hyperspectral observations were conducted of the winter wheat crop at the Langfang Experimental Station, Institute of Plant Protection, Chinese Academy of Agricultural Sciences (39°30′42″N, 116°36′07″E) in Hebei Province, China from April to May 2018. A winter wheat cultivar, “Mingxian 169”, which is highly susceptible to yellow rust, was used in the experiment. It was sown on 4 October 2017 and was inoculated with yellow rust pathogen (9 mg/100 mL) on 12 April by artificial inoculation. The experimental field contained one control group and two fields were used for experimenting with wheat infected with yellow rust. Each field occupied an area of approximately 3 × 16 m^2^. Each field had 8 sample plots, and each sample plot with an area of 1 m^2^ were selected in the field for canopy spectral measurement. For the control group and the groups infected by yellow rust, the canopy spectral measurements were repeated 8 and 16 times, respectively. All groups were prepared similarly when sowing took place (200 kg/ha nitrogen and 450 m^3^/ha water) and were subsequently managed under the same conditions.

### 2.2. Canopy Spectral Measurements

The spectral reflectance of the canopy was collected with an ASD FieldSpec spectrometer (Analytical Spectral Devices, Boulder, CO, USA). The spectrometer was fitted with a 25° field-of-view fore optic in the spectral range between 350 nm and 2500 nm. The spectral resolution was 3 nm and 10 nm in the 350–1000 nm and 1000–2500 nm ranges, respectively. All canopy spectral measurements were taken at a height of 1.3 m above the ground. A 40 cm × 40 cm BaSO_4_ calibration panel was measured to correct the reflectance. The spectrum of each sample was measured 20 times and then the mean was used as the reflectance spectrum. All spectral reflectance measurements were collected between 10:00 and 14:00 (Beijing local time) under cloudless conditions.

In Experiment 1, the spectral measurements were recorded 5 times, starting from 207 days after sowing (DAS) and ending 239 DAS. This period included the jointing stage (207 DAS), booting stage (216 DAS), anthesis stage (225 DAS), filling stage (230 DAS), and milky ripeness stage (238 DAS). In each growth stage of winter wheat, 31 spectral reflectance samples were selected (5 healthy samples and 26 infected samples) with different severity levels of yellow rust for further study. In Experiment 2, the canopy spectral reflectance was obtained during the major growth stages consistent with those in Experiment 1, from the jointing to the milky ripeness stage. For experiment 2, 7 healthy and 15 infected samples were selected in each growth stage. In particular, the severity of the disease pathogen and the growth stages were also recorded.

### 2.3. Assessment of Disease Index

The disease index (DI) was used to describe the severity of crop diseases [2]. In each plot, 40 wheat plants were randomly selected to measure the incidence of wheat yellow rust. Human error was eliminated by ensuring that all incidents of yellow rust disease were assessed by the same person under the guidance and supervision of a professional who majored in plant protection. According to the National Rules for the Investigation and Forecasting of Crop Diseases (GB/T 15795-1995), the formula for calculating the DI is as follows [22]:

(1) $$DI=\frac{\sum xf}{n\sum f}\times 100$$

where *x* is the value of the incidence level, *n* is the value of the highest disease severity gradient (*n* = 8), and *f* is the number of leaves for each degree of disease severity. 

### 2.4. Commonly Used Spectral Indices in YR Detection

Spectral indices are widely used for monitoring, analyzing, and mapping temporal and spatial variation in vegetation [27]. Spectral indices are the basis for many applications of remote sensing in crop management because they are highly correlated to biophysical and biochemical crop variables [28]. As pigment content provides information on the physiological state of leaves, pigment-specific vegetation indices may be also useful in detecting the amount of stress caused by fungal diseases. The efficiency of spectral indices to identify and discriminate between healthy and infected yellow rust disease can be evaluated by calculating the vegetation indices related to different physiological parameters (Table 1). This list of 15 indices related to hyperspectral indices was compiled from the literature and they were further tested for their ability to detect yellow rust infection in this study. These indices are the Structural Independent Pigment Index (SIPI), Photosynthetic Radiation Index (PRI), Transformed Chlorophyll Absorption in Reflectance Index (TCARI), Normalized Difference Vegetation Index (NDVI), Normalized Pigment Chlorophyll Index (NPCI), Plant Senescence Reflectance Index (PSRI), Physiological Reflectance Index (PhRI), Anthocyanin Reflectance Index (ARI), Modified Simple Ratio (MSR), Ratio Vegetation Structure Index (RVSI), Modified Chlorophyll Absorption Reflectance index (MCARI), Yellow Rust Index (YRI), Greenness index (GI), Triangular Vegetation Index (TVI), and Nitrogen Reflectance Index (NRI).

### 2.5. Testing the Performance of Vegetation Indices

In this study, linear regression was employed to model the relationship between indices (existing spectral indices and optimized spectral indices) and yellow rust disease index at different growth stages and to validate the models. The coefficient of determination (R^2^) was used to evaluate the performance of spectral indices. 

In addition, the linear discrimination analysis (LDA) model was used for testing and evaluating the performance of the vegetation indices in terms of detecting yellow rust disease. Linear discriminant analysis uses the non-parametric K-means clustering method to establish a classification model and has been widely used in the classification of crop diseases [2,6]. In the model, the actual measured disease index (DI) was used as samples for training and evaluating the spectral indices at different growth stages. In consideration of practical application, the disease index of the canopy was quantitatively classified into two classes, healthy and diseased, for modeling. Leave-one-out cross validation was applied to verify the classification accuracy, this means that each sample was used as validation sample in this model, and the other *N*-1 sample was used as training sample (*N* is the total number of samples), Finally, *N* models will be obtained, and the average of the classification accuracy of the validation samples of the *N* models was used as a performance criterion to the LDA model, specifically, the overall accuracy, producer’s accuracy, user’s accuracy, and kappa coefficient were used to evaluate the LDA model from different aspects. The LDA model was implemented using SPSS 20.0 software (IBM Corporation, New York, NY, USA).

## 3. Results

### 3.1. Canopy Spectral Reflectance of Wheat Yellow Rust Disease at Different Growth Stages 

**Figure 1:**
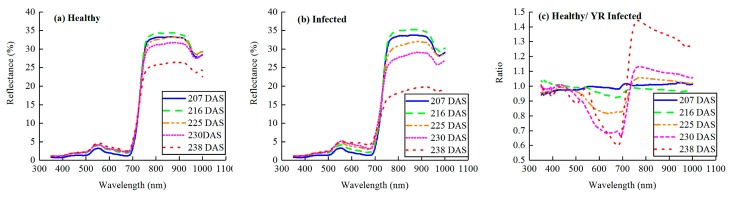
Average reflectance curves of (**a**) healthy wheat and (**b**) wheat infected by yellow rust; (**c**) spectral ratio of healthy and yellow rust-infected wheat at five stages starting on the day of sowing until 207, 216, 225, 230, and 238 days after sowing.

The spectral response properties of the wheat canopy to fungi stress were very important for discriminating yellow rust infection levels in precise disease management using hyperspectral remote-sensing data. The average canopy reflectance spectra of healthy and yellow rust infected at five growth stages are shown in Figure 1. Healthy green plants have high absorption in the visible region except in the green band, whereas in the infrared region the reflectance was high [40]. During these periods, the shape of the reflectance curve of the healthy wheat canopy and that infected by yellow rust was basically similar in that it remained constant with strong absorption by photosynthetic pigments in the visible region and exhibited a high reflectance plateau in the near infrared region (Figure 1a,b). Generally, with the increasing growth stage, the reflectance of trend to increase in the visible region and initially increased then decreased in the near-infrared region (Figure 1a) [41]. 

Compared with the healthy wheat, the spectral reflectance of the yellow rust infected wheat was increased in visible light, while it decreased in the near-infrared region at the same growth stage (Figure 1a,b). The differences of spectral reflectance magnitude between healthy and infected wheat were not obvious in the 207 DAS and 216 DAS during jointing stage and heading stage, but it was distinct in the late stages when sowing days (Figure 1c). During 225 DAS, 230 DAS, and 238 DAS, yellow rust-infected wheat had a higher visible reflectance from 500 nm to 700 nm than healthy wheat, and the reflectance in the near infrared region was lower than healthy wheat. From the perspective of growth period, the spectral differences showed a stronger response in 520–710 nm and 730–1000 nm at 238 DAS than 207 DAS for both healthy and yellow rust-diseased samples (Figure 1c), especially in the red region and near infrared region. This change might relate to the content of pigments in mesophyll tissue and the senescence process of leaves [42]. The dynamic change patterns of canopy spectral reflectance under different growth stages of winter wheat provided a basis for analyzing and construction quantitative relation of DI to canopy spectral reflectance characteristics in wheat.

### 3.2. Sensitive Regions to Discrimination of Wheat Yellow Rust in Different Growth Stages

**Figure 2:**
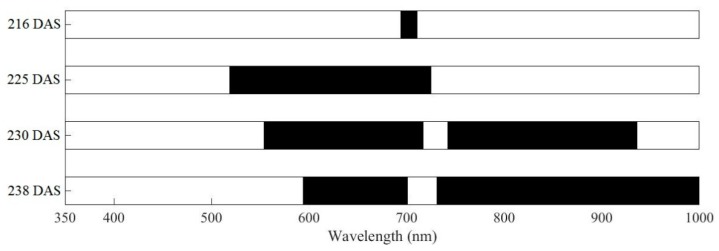
Wavelengths of correlation between canopy reflectance and yellow rust disease index at a 0.999 confidence level for four different growth stages.

An independent *t*-test was applied to examine the statistical significance of healthy and infected samples in the range 350–1000 nm of the five growth stages. The spectral bands that were significant in the response (*p* < 0.001) are shown in Figure 2. The correlation analysis of the growth stage until 207 DAS was not obvious, because the symptoms of yellow rust disease had not yet emerged during this period (corresponding to the jointing stage). The green, red, and red-edge wavebands in the visible region are sensitive to wheat yellow rust discrimination in the four other growth stages; in addition, the near infrared region wavebands were also sensitive to disease discrimination in the periods 230 DAS and 238 DAS. The sensitive bands were selected by conducting correlation analysis between the spectral reflectance and yellow rust disease index in these four growth stages. The sensitive bands were in the regions 694–711 nm and 519–720 nm in the periods 216 DAS (corresponding to the booting stage) and 225 DAS (corresponding to the anthesis stage), respectively. The bands sensitive to yellow rust discrimination were located in the regions 554–717 nm and 772–936 nm for the period 230 DAS (corresponding to the filling stage), whereas for 238 DAS (corresponding to the milky ripeness stage) they were located in 594–701 nm and 731–1000 nm. These results indicated that there were differences among the different growth stages in terms of spectral bands for yellow rust monitoring, although some of the bands were common to more than one growth stage.

The sensitive regions of 216 DAS and 225 DAS were located in the visible region, whereas those of 230 DAS and 238 DAS were located in the visible and near infrared regions. In practice, the growth of healthy wheat was terminated by restricting the supply of nutrient substances in late growth period. This leads to similarity of the spectral signatures between healthy wheat at the late growth stage and infected wheat at the early growth stage. The amount of spectral information relating to disease infection by growth state was reduced by evaluating the four different growing stages as two main stages, namely the early-mid stage, which includes the jointing and booting stages (216 DAS and 225 DAS), and the mid-late stage, which includes the filling and milky ripeness stages (230 DAS and 238 DAS).

**Figure 3:**
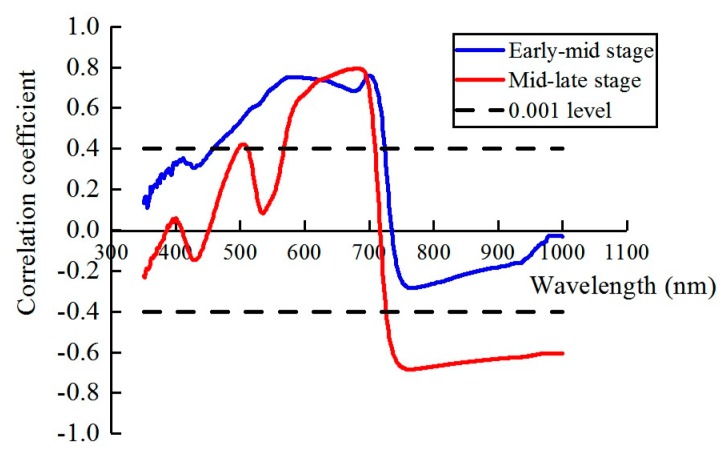
Correlation coefficient between reflectance and yellow rust disease index in the two main growth stages.

To undertake an initial first pass on the wavebands, correlation analysis was used to assess whether significant relationships existed between the mean canopy spectra and the yellow rust disease index of wheat. In the early-mid stage, the green, red, and red-edge region (460–720 nm) showed high correlation, especially in the red-edge and green regions. In the mid-late stage, part of the green, red, and red-edge region and the near infrared bands (568–709 nm and 727–1000 nm) showed a high correlation with wheat infected by yellow rust, especially in red-edge and near infrared regions (Figure 3). This is attributed to the time lag between rust infection and internal structural damage during the early stage of yellow rust infection [4], causing the near infrared region to be insensitive in this stage but sensitive in the mid-late growth stage. Table 2 summarizes the information about the disease index (DI) of wheat yellow rust under different growth stages in different experiments according to Section 2.3.

### 3.3. Response of Existing Spectral Indices to Yellow Rust at Different Growth Stages 

Spectral indices have been developed to estimate different plant parameters using a few wavelengths. Table 3 summarizes the responses of all spectral indices to yellow rust disease at different growth stages and in all growth stages. We found that these selected 15 indices manifested excellent potential for discriminating yellow rust disease throughout the growth stage (*p* < 0.001), except for TCARI (*p* < 0.01). However, not all spectral indices selected were all able to significantly discriminate wheat yellow rust in each growth stages. The response of the vegetation indices to yellow rust at different growth stages differs from that of all growth stages; for example, TCARI, PhRI, RVSI, and MCARI are sensitive to yellow rust discrimination and are insensitive to the mid-late growth stage, and YRI showed a significant response in the mid-late growth stage but is insensitive to the early-mid growth stage. Of these indices, PRI showed the highest correlation of determination with R^2^ = 0.65 at the early-mid growth stage, and ARI showed good relationships with yellow rust disease with R^2^ = 0.81 at the mid-late growth stage. The response of the same index in different growth stages to yellow rust disease is different and is mainly affected by the mechanism whereby vegetation becomes disease-infected [4]. At all growth stages, the two indices ARI and PRI showed higher coefficients of determination (R^2^ = 0.85 and 0.83, respectively). This shows that ARI and PRI can effectively identify wheat yellow rust disease, which is consistent with the research of Devadas et al. [10] and Huang et al. [22]. Among these indices, SIPI, PRI, NDVI, PSRI, ARI, MSR, GI, and NRI showed excellent potential for discriminating yellow rust disease in different growth stages (*p* < 0.001) and they were used for subsequent study.

### 3.4. Construction of the Vegetation Index

Sensitive bands containing mainly spectral information of crop variables are the foundation and precondition for constructing a spectral index [41]. It is known from Section 3.2 that in different growth stages, the spectral regions that are sensitive to wheat affected by yellow rust are different, with the sensitive bands occurring across a wide region. However, PRI is composed of two green bands, and ARI is composed of green and red-edge bands, and are the best vegetation spectral indices for monitoring wheat diseases. Inspired by this information, we attempted to propose indices that combine three-band indices in the form of PRI and ARI in the wavelength ranges that are sensitive to wheat with yellow rust disease. The new three-band indices are expressed as Equations (2) and (3).

(2) $$PRI(\lambda 1,\lambda 2,\lambda 3)=\frac{R_{\lambda 1}-R_{\lambda 2}}{R_{\lambda 1}+R_{\lambda 3}}$$

(3) $$ARI(\lambda 1,\lambda 2,\lambda 3)=\frac{1}{R_{\lambda 1}-R_{\lambda 2}}-\frac{1}{R_{\lambda 2}-R_{\lambda 3}}$$

where *R_λ_*_1_, *R_λ_*_2_, and *R_λ_*_3_ are the spectral reflectance of random wavelengths from the sensitive bands in Section 3.2 at different growth stages, and λ1 ≠ λ2 ≠ λ3.

**Figure 4:**
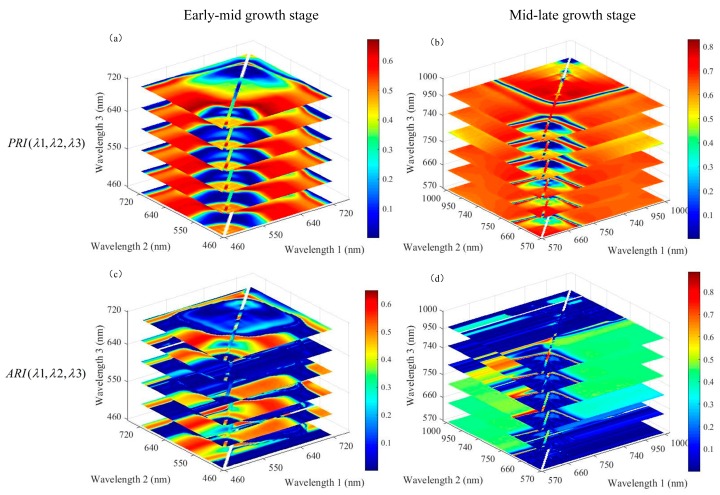
Three-dimensional slice maps of the correlation coefficients (R^2^) for the relationships between the yellow rust disease index and the new indices calculated from all possible three-band combinations at 3 nm band sampling intervals from the wavelengths sensitive in the different growth stages. (**a**) and (**b**) R^2^ of PRI (λ1, λ2, λ3) (Photochemical Reflectance Index) at the early-mid and mid-late growth stages, respectively; (**c**) and (**d**) R^2^ of ARI (λ1, λ2, λ3) (Anthocyanin Reflectance Index) at the early-mid and mid-late growth stages, respectively; λ1, λ2, and λ3 are the wavelengths sensitive to yellow rust discrimination.

These indices were designated using all possible band combinations available for the sensitive wavelength ranges. A large number of indices provide many opportunities to study the changes in the biophysical and biochemical characteristics of a crop subjected to stress. However, hyperspectral narrowband data offer high correlation between adjacent bands, and using all the bands would only increase the computational complexity without adding additional information. In addition, the best information is contained in only a few selected bands and the remainder becomes redundant [43]. According to studies by Thenkabail et al. [43], maintaining the narrowband width at 3 nm achieves optimal results in the quantitative modeling of agricultural crops. Therefore, to enable us to select the optimal index for detecting yellow rust, combinations of all possible spectral indices in both of the above forms with a bandwidth of 3 nm were proposed for subsequent analysis of different stages. In this study, the yellow rust disease index was set as the independent variable and all possible spectral indices as the dependent variable. Then, quantitative regression analysis was performed to investigate the relationship between the yellow rust disease index and combinations of all the bands of the new three-band indices in different growth stages. The coefficients of determination (R^2^) were calculated and adopted as the comprehensive index to evaluate the ability of spectral indices to invert wheat yellow rust severity. Figure 4 shows three-dimensional slice maps of the R^2^ values for estimation of the yellow rust disease index in different growth stages with the two types of proposed three-band spectral indices, respectively. The *x*-, *y*-, and *z*-axes represent the wavelength regions that are sensitive to the discrimination of yellow rust disease. The aforementioned procedures were implemented by self-programming using MATLAB software.

According to the correlation coefficient (R^2^) of all possible indices and the yellow rust disease index, the maximum value of PRI (λ1, λ2, λ3) (R^2^ = 0.672) was higher than that of ARI (λ1, λ2, λ3) (R^2^ = 0.651) in the early-mid growth stages, whereas the maximum value of PRI (λ1, λ2, λ3) (R^2^ = 0.828) was lower than that of ARI (λ1, λ2, λ3) (R^2^ = 0.889) in the mid-late growth stages in Figure 4. In this study, the three-band spectral indices with a large correlation coefficient were selected for subsequent research on the identification of wheat affected by yellow rust disease in the same growth stage. Therefore, the optimal vegetation index in the early-mid growth stage is the combination of the three bands of PRI (λ1, λ2, λ3), which correspond to the maximum correlation coefficient, and the best index is the three-band spectral index of ARI (λ1, λ2, λ3) for the mid-late growth stage. 

In the early-mid growth stage, all three wavelengths of the three-band optimal PRI (λ1, λ2, λ3) of the index exploited the green and red-edge bands. In the mid-late growth stage, all wavelengths of the three-band optimal ARI (λ1, λ2, λ3) of the index consisted of the red-edge and near infrared bands. The three-band optimized indices composed of the central spectral bands in the zones with the highest R^2^ were selected [44]. Therefore, the bands of 570 nm, 525 nm, and 705 nm were selected in the early-mid growth stage, and the bands of 860 nm, 790 nm, and 750 nm were selected in the mid-late growth stage. Finally, the three-band indices of PRI (570, 525, 700) and ARI (860, 790, 750) are proposed as the optimal indices for the discrimination of wheat yellow rust in the early-mid and mid-late growth stages, respectively.

### 3.5. Performance of New Spectral Indices for the Two Main Growth Stages

#### 3.5.1. Comparison of the Relationship Between the Yellow Rust Disease Index and Selected Spectral Indices

**Figure 5:**
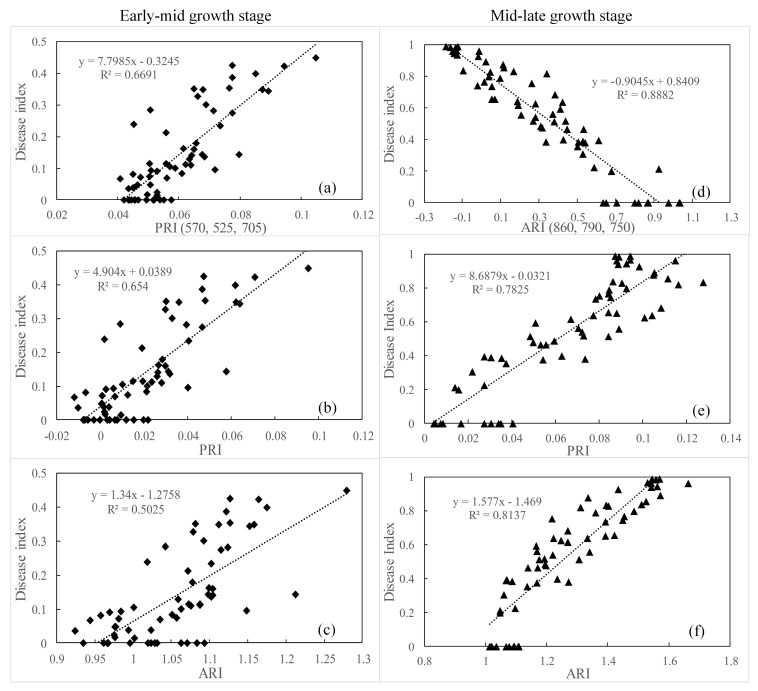
Scatter plots of the relationships between the three-band spectral indices and corresponding best performing published indices versus the yellow rust disease index at different growth stages. Relationship between the spectral indices and yellow rust disease index in the (**a–c**) early-mid growth stage and (**d–f**) mid-late growth stage.

The best performing published spectral indices for yellow rust monitoring in previous studies (Section 3.2) were tested against the new indices proposed in this study. Figure 5 shows scatter plots of the relationship between the severity of yellow rust and the new indices and commonly used vegetation indices for the different growth stages. In this study, the PRI (570, 525, 705) was linearly related to the yellow rust disease index in the early-mid growth stage and produced the highest R^2^ value of 0.669, which is higher than that of the best-performing published index PRI (R^2^ = 0.65) and ARI (R^2^ = 0.50). The coefficient of determination of the linear regression model for ARI (860, 790, 750) and the yellow rust disease index is 0.89 in the mid-late growth stage, which is 0.11 and 0.08 higher than PRI and ARI, respectively. This suggested that PRI (570, 525, 705) and ARI (860, 790, 750) greatly improve the accuracy of estimation of the yellow rust disease index of wheat, which is highly sensitive for the early-mid and mid-late growth stages of the canopy, respectively. 

#### 3.5.2. Ability of New Spectral Indices to Discriminate Yellow Rust Disease at Different Growth Stages

In the actual analysis, for a disease index less than 0.05, the canopy spectrum of healthy and infected samples is similar. Therefore, samples of which the DI is less than 0.05 were considered to be healthy samples, and the differentiation between healthy wheat and that infected by yellow rust was used to assess and compare the effectiveness of the two new indices for different growth stages. The ability of PRI (570, 525, 705) and ARI (860, 790, 750) to discriminate between healthy and infected wheat at different growth stages is summarized in Table 4 based on the linear discrimination analysis (LDA) model. The overall classification accuracy and kappa coefficient of PRI (570, 525, 705) was 80.6% and 0.61 for the early-mid growth stage. In the mid-late growth stage, these values were 91.9% and 0.75 for ARI (860, 790, 750).

The linear discrimination analysis (LDA) model [45] was used to further test the two new spectral indices that were developed using sensitive band combinations along with other common spectral indices for crop disease stress. These existing indices, which were obtained from the literature, were selected for their predictability of the severity of yellow rust in plants sampled across the different growth stages (Table 5). In the early-mid growth stage, PRI (570, 525, 705) achieved the best classification accuracy (80.6%) for the discrimination between healthy and wheat infected by yellow rust, followed by PRI and ARI with an overall classification accuracy of 79.0%. In the mid-late growth stage, ARI (860, 790, 750) achieved the best classification accuracy (91.9%) for the discrimination between healthy and wheat infected by yellow rust, followed by PRI and NPCI with an overall classification accuracy of 87.5% and 79.0%. Both the PRI (570, 525, 705) and ARI (860, 790, 750) have excellent performance in monitoring yellow rust in wheat compared with the existing spectral indices for the early-mid and mid-late growth stages, respectively. The model based on PRI (570, 525, 705) and ARI (860, 790, 750) significantly increased the classification accuracy of healthy wheat and that infected by yellow rust at the early-mid and mid-late growth stages with the canopy, respectively.

### 3.6. Testing New Spectral Indices on a Different Database 

**Figure 6:**
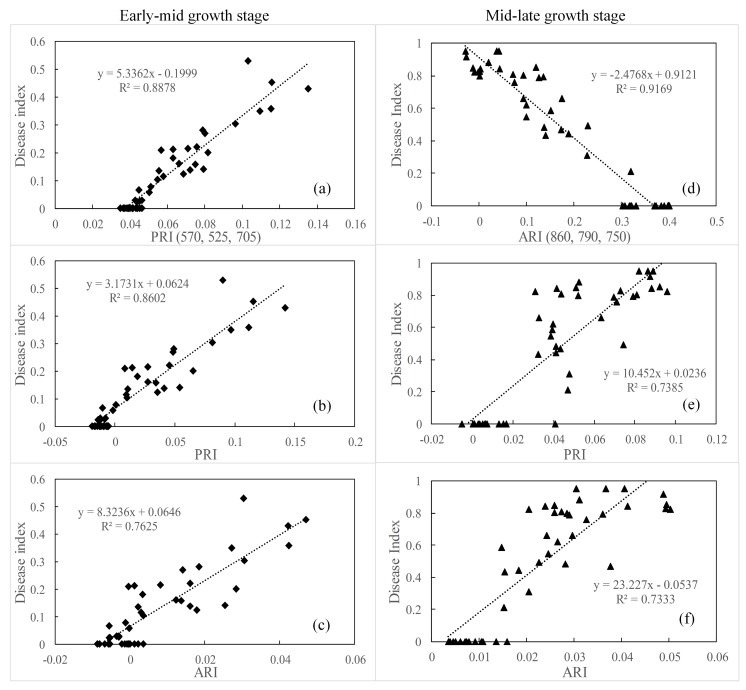
Scatter plots of the relationships between the three-band spectral indices and corresponding best-performing published indices versus the yellow rust disease index for the different growth stages in the validating database.

To further validate the ability of PRI (570, 525, 705) and ARI (860, 790, 750) to detect yellow rust disease, independent data were used in Experiment 2. Scatter plots of the relationship between the severity of yellow rust disease and the best-performing indices of PRI (570, 525, 705) and ARI (860, 790, 750) are shown in Figure 6 for the different growth stages. The PRI (570, 525, 705) showed an R^2^ value of 0.89 in the early-mid growth stage, and ARI (860, 790, 750) showed an R^2^ value of 0.92 in the mid-late growth stage, respectively, which generated a higher coefficient than PRI and ARI in the corresponding growth stage. This indicates that the regression model with PRI (570, 525, 705) and ARI (860, 790, 750) has the apparently stability to estimate the severity of yellow rust disease in the early-mid and mid-late growth stages, respectively.

Table 6 shows the ability of PRI (570, 525, 705) and ARI (860, 790, 750) to discriminate between healthy wheat and wheat infected by yellow rust. The PRI (570, 525, 705) showed a higher classification accuracy of wheat infected by yellow rust with an overall classification accuracy of 84.1% compared with the existing spectral indices for the early-mid growth stage, i.e., 2.3% and 4.6% higher than PRI and ARI, respectively. For the mid-late growth stage, ARI (860, 790, 750) achieved the best classification accuracy (up to 93.2%) for discriminating between healthy wheat and that infected by yellow rust, with the classification accuracy of healthy wheat being as high as 100.0% and that of wheat infected by yellow rust reaching 90.0%, which are higher than the results obtained for existing spectral indices. Consequently, the new indices for yellow rust monitoring were tested with independent data and the validation indicated that the indices performed well for experimental data from different years, wheat cultivars, and sites. Then, PRI (570, 525, 705) and ARI (860, 790, 750) performed reliably in terms of detecting wheat infected by yellow rust disease in the early-mid and mid-late growth stages, respectively.

## 4. Discussion

Monitoring crop disease throughout all the growing stages provides a favorable basis for guiding the precise management of agriculture by guiding the site-specific application of fungicide for disease prevention in the early growth stage, as well as assess the yield loss in the later stage [46,47]. The most obvious symptoms of winter wheat infected with stripe rust were green fading and the deformation of leaf tissue, which change the morphological and physiological parameters such as biomass, chlorophyll level, and water content, and also cause changes in their corresponding spectra [21,48]. The difference in the spectral response of infected and healthy wheat is the basis of optical diagnostics for crop diseases. In Figure 2, the sensitive wavelength regions that enable yellow rust disease to be discriminated differ for different growth stages. At the early stage of infection, yellow rust funguses tend to reproduce in large quantities, and the physiological and biochemical characteristics of infected plants are not obvious [8]. Thereby, the sensitive wavelength range of yellow rust discrimination in the booting stage is located in the region 694–711 nm. As the disease develops, disease pathogens can induce changes in the biophysical and biochemical parameters of plants, such as variations of several pigments, water content, and canopy structure [47]. In addition, leaf color changes due to pustules or lesions cause the physiological and biochemical characteristics of plants to change under the influence of diseases [4,49], which in turn changes the response of a series of spectral features. Furthermore, the sensitive wavelength range of the filling and milky ripeness stages are located in the visible and near infrared regions. This is because of the time lag between rust infection and the breakdown of the internal leaf structure in the early-mid growth stage of yellow rust infection research. Infected wheat in the early-mid stage is not sensitive to near infrared region, contrary to the mid-late stage [4].

According to previous studies related to the remotely-sensed detection of wheat infected by yellow rust disease, we found that ARI and PRI were reported as efficiently vegetation indices for yellow rust disease monitoring at the canopy scale [4,22], which was consistent with our results. It should be noted that PRI was used to track changes in the photosynthetic efficiency, which is the normalized form of the reflectance of 570 nm and 531 nm [30]. Based on the form of PRI, by combining all three possible bands over the sensitive wavelengths, we were able to select the three bands of 570 nm, 525 nm and 705 nm to construct the spectral index PRI (570, 525, 705) with sensitivity to the discrimination of yellow rust disease in the early-mid growth stage. The bands 570 nm and 525 nm lie in the green region. Wheat is infected by yellow rust disease, in which case pathogens interfere with their photosynthesis by affecting the chloroplasts and cause their degeneration. This induces strong spectral responses, which are closely linked to the green band in the visible region [21]. According to Xue et al. [50], the band centered at 526 nm is often the result of strong absorption by plant chlorophyll and carotenoids for photosynthetic production, and can be considered as representative of green plant photosynthesis. The band at 570 nm appears in both the three-band and two-band spectral indices of PRI. Wavelengths from the green bands were also used for discriminating wheat diseases [22]. Thus, these bands are critical for the discrimination of crop infected by disease. The band at 705 nm is located in the red-edge region and is an indicator of plant stress [24]. A similar result was reported by Moshou et al. [1], where the reflectance of the band centered at 705 nm is sensitive to the detection of wheat infected by yellow rust. In this study, the accuracy of the model (80.6%) for PRI (570, 525, 705), which is 1.6% higher than that of the best-performing published index PRI, suggested that PRI (570, 525, 705) is highly sensitive for detecting yellow rust disease in the early-mid growth stage (Table 5). 

The ARI (860, 790, 750) was constructed by the wavebands of 860 nm, 790 nm, and 750 nm in the form of ARI, which offered excellent detection of wheat infected by yellow rust disease in the mid-late growth stage. In the mid growth stage, the rust spores cover the wheat leaves when the disease is at its peak. This causes the leaves to roll up and rupture the foliar epidermis. The pathogenic infection changes the canopy density and leaf area, and the near infrared region is a sensitive indicator for changes in the canopy structure [51,52]. The bands at 860 nm and 790 nm, located in the near infrared region, can be used to predict the potential differences in photosynthesis and changes in the canopy structure [50]. In addition, according to Feng et al. [21], spectral wavebands in the red-edge region (700–750 nm) are regarded as useful parameters to indicate the status of vegetation nutrition, growth, moisture, and the leaf area. The band at 750 nm is located in the red-edge region and is sensitive to discriminate yellow rust disease in the mid-late growth stage. This was in agreement with research using the wavebands at approximately 750 ± 10 nm and 861 ± 10 nm that were also used by Bravo et al. [13] to discriminate wheat infected by yellow rust from healthy wheat. It was concluded that red-edge wavelengths should be useful in reflectance studies of crop disease throughout out the season. The results of this study agree with those reported by Yu et al. [53], who pointed out that the hyperspectral narrowband of the red-edge in the near infrared region was identified as effective bands for disease discrimination in vegetation. In this study, the accuracy of the model (R^2^ = 0.888) for ARI (860, 790, 750), which is 0.075 higher than that of the best-performing published index ARI, suggested that ARI (860, 790, 750) is highly sensitive for estimating the severity of yellow rust in the mid-late growth stage (Figure 5).

In this study, we explored the ability of three-band vegetation indices to discriminate wheat infected by yellow rust in different growth stages according to the best-performing form of the spectral vegetation index. The PRI (570, 525, 705) enriches the red-edge information of a crop under disease stress compared with PRI. This is in accordance with the idea that the red-edge wavelength band is the most sensitive to differences between infected and healthy wheat. Moreover, ARI (860, 790, 750) not only takes into account the red-edge information, but also the influence of structural changes in the canopy because of vegetation infection disease in the mid-late growth stage. The coefficient of determination of PRI (570, 525, 705) is 0.669 for estimating yellow rust disease in the early-mid growth stage. This is lower than for ARI (860, 790, 750) in the mid-late growth stage (R^2^ = 0.888) in Figure 5. During different growth stages, it remains difficult to estimate disease severity during the early growth period, which is due to the underdevelopment of the disease and the resulting spectral similarity between wheat infected with yellow rust and healthy wheat. However, in the mid-late stage, yellow rust disease could be distinguished more easily than in the early-mid stages, and the classification accuracy is up to 91.9% in Table 4. These results are consistent with the study of Sanakran et al. [47] who indicated that the higher the visible symptoms, the more accurate the disease detection. Based on these results, the indices of more stable structures can be explored for their ability to discriminate wheat yellow rust disease in future research, such as geometric forms and the area form. Furthermore, the canopy reflectance of healthy and infected wheat at different growth stages and under different degrees of disease severity were collected using a hand-held hyperspectrometer in the spectral range of 350–1000 nm that was used in this study. In the visible wavelength range (400–700 nm), changes in the leaf pigment can be captured, whereas the near infrared region (700–1000 nm) is related to the status of the cell structure [54]. However, changes in the plant water content are extended to the shortwave infrared region (1300–2500 nm), a spectral range that will also be considered in future research for the detection of wheat infected by yellow rust disease. 

## 5. Conclusions

The timely monitoring of wheat infected by yellow rust disease is critical for agricultural management of the growth stage. Based on the growth status of wheat and the wavelength regions that are sensitive to wheat infected by yellow rust in all growth stages, we divided the entire growth stage into two main stages, i.e., the early-mid growth stage (the jointing and booting stages) and the mid-late growth stage (the filling and milky ripeness stages). The wavebands sensitive to the discrimination of wheat infected by yellow rust are located in the visible region for the early-mid growth stage, and in both the visible and near infrared regions for the mid-late growth stage, respectively. Two three-band spectral indices, PRI (570, 525, 705) for the early-mid stage and ARI (860, 790, 750) for the mid-late growth stage, were recommended as the best spectral indices for monitoring yellow rust disease in wheat. The PRI (570, 525, 705) can more effectively estimate the severity of yellow rust disease with an R^2^ of 0.669, and its classification accuracy of healthy and yellow rust infected wheat reached 80.6% during the early-mid growth stage. The ARI (860, 790, 750) estimated the severity of yellow rust disease during the mid-late growth stage with higher precision (R^2^ = 0.888), and the classification accuracy of healthy and yellow rust infected wheat reached 91.9%. Furthermore, the two novel spectral indices for the discrimination of yellow rust infection proved accurate for different wheat varieties and environmental conditions in different growth stages. Nevertheless, additional studies are needed to confirm the universality of the two new indices for different wheat cultivars and fields. The timely discrimination of yellow rust disease in wheat is critical for maximizing yield and minimizing adverse environmental impacts, and to provide data for the local agricultural insurance services. Hence, in the future, we will consider applying the indices to monitoring wheat infected by yellow rust disease for different disease severities, rather than simply identifying healthy and infected wheat. Subsequently, the indices could be applied to hyperspectral airborne or space-borne imagery for monitoring wheat infected by yellow rust disease in larger field regions in future research.

## Figures and Tables

**Table 1:** Published spectral indices tested in this study.

Define	Formula	Related to	Reference
Structural Independent Pigment Index, SIPI	(R_800_ − R_445_)/(R_800_ − R_680_)	Pigment content	[4,29]
Photochemical Reflectance Index, PRI	(R_570_ − R_531_)/(R_570_ + R_531_)	Photosynthetic radiation	[30,31]
Transformed Chlorophyll Absorption in Reflectance Index, TCARI	3((R_700_ − R_675_) − 0.2(R_700_ − R_500_)/(R_700_/R_670_))	Chlorophyll a + b concentration	[32]
Normalized difference vegetation index, NDVI	(R_830_ − R_675_)/(R_830_ + R_675_)	Leaf area index; photosynthetically active radiation (PAR) or biomass (PAB)	[16]
Normalized Pigment Chlorophyll Index, NPCI	(R_680_ − R_430_)/(R_680_ + R_430_)	Chlorophyll ratio	[4]
Plant senescence reflectance index, PSRI	(R_680_ − R_500_)/R_750_	Pigment content; leaf senescence and ripening	[17]
Physiological Reflectance Index, PhRI	(R_550_ − R_531_)/(R_550_ + R_531_)	Light use efficiency	[30]
Anthocyanin Reflectance Index, ARI	(R_550_)^−1^ − (R_700_)^−1^	Anthocyanin content	[10,33]
Modified Simple Ratio, MSR	(R_800_/R_670_ − 1)/sqrt(R_800_/R_670_ + 1)	Leaf area	[34]
Ratio Vegetation Structure Index, RVSI	(R_712_ + R_752_)/2) − R_732_	Internal structure parameter	[35]
Modified Chlorophyll Absorption Reflectance index, MCARI	((R_701_ − R_671_) − 0.2(R_701_ − R_549_))/(R_701_/R_671_)	Chlorophyll absorption	[36]
Yellow rust index, YRI	(R_730_ − R_419_)/(R_730_ + R_419_) + 0.5R_736_	Wheat disease	[31]
Greenness index, GI	R_554_/R_677_	Pigment content	[37]
Triangular vegetation index, TVI	0.5(120(R_750_ − R_550_) − 200((R_670_ − R_550_))	Plant status	[38]
Nitrogen reflectance index, NRI	(R_570_ − R_670_)/(R_570_ + R_670_)	Nitrogen status	[39]

**Table 2:** The range value of disease index (DI) and corresponding sample size for different severity levels of disease in different growth stages and different experiments.

Experiments	DI Range	Number of Samples in Different Growth Stages	
		Early-Mid Growth Stage	Mid-Late Growth Stage
Experiment 1 (2003)	DI: <5%	21	10
	DI: 5–30%	29	3
	DI: >30%	12	48
Experiment 2 (2018)	DI: <5%	22	14
	DI: 5–30%	17	1
	DI: >30%	5	29

**Table 3:** Response of spectral indices and yellow rust disease index at different growth stages.

Spectral Indices	Response to Yellow Rust at Different Growth Stages		
	Early-Mid Growth Stage	Mid-Late Growth Stage	All Growth Stage
SIPI	0.52 ***	0.60 ***	0.67 ***
PRI	0.65 ***	0.78 ***	0.83 ***
TCARI	0.30 ***	0.005	0.07 *
NDVI	0.40 ***	0.62 ***	0.72 ***
NPCI	0.52 ***	0.79 **	0.80 ***
PSRI	0.53 ***	0.68 ***	0.74 ***
PhRI	0.48 ***	0.07	0.19 ***
ARI	0.50 ***	0.81 ***	0.85 ***
MSR	0.36 ***	0.68 ***	0.73 ***
RVSI	0.42 ***	0.05	0.40 ***
MCARI	0.58 ***	0.06	0.29 ***
YRI	0.001	0.36 ***	0.47 ***
GI	0.29 ***	0.71 ***	0.67 ***
TVI	0.10 **	0.54 ***	0.64 ***
NRI	0.27 ***	0.65 ***	0.66 ***

* Indicates the correlation is significant at the 0.950 confidence level. ** Indicates the correlation is significant at the 0.990 confidence level. *** Indicates the correlation is significant at the 0.999 confidence level.

**Table 4:** Overall results of the discriminant model based on the new indices for identifying healthy wheat and that infected by yellow rust for the different growth stages.

**Early-Mid Growth Stage**						
PRI (570, 525, 705)		**Healthy**	**Yellow rust**	**U (%)**	**OAA (%)**	**Kappa**
	Healthy	20	11	64.5	80.6	0.61
	Yellow rust	1	30	96.8		
	P (%)	95.2	73.2			
**Mid-Late Growth Stage**						
ARI (860, 790, 750)		**Healthy**	**Yellow rust**	**U (%)**	**OAA (%)**	**Kappa**
	Healthy	10	5	66.7	91.9	0.75
	Yellow rust	0	47	100.0		
	P (%)	100.0	90.4			

Note: P = producer’s accuracy, U = user’s accuracy, OAA = overall accuracy.

**Table 5:** Comparison of the ability of PRI (570, 525, 705), ARI (860, 790, 750), and common indices to discriminate between healthy wheat and wheat infected by yellow rust at different growth stages.

Index	Early-Mid Growth Stage (216 DAS, 225 DAS)			Mid-Late Growth Stage (230 DAS, 238 DAS)		
	Overall Classification Accuracy (%)	Recall		Overall Classification Accuracy (%)	Recall	
		Healthy (%)	Yellow Rust (%)		Healthy (%)	Yellow Rust (%)
PRI (570, 525, 705)	80.6	95.2	73.2	/	/	/
ARI (865, 790, 750)	/	/	/	91.9	100.0	90.4
PRI	79.0	90.5	73.2	87.5	100.0	84.6
ARI	79.0	81.0	78.0	77.4	100.0	73.1
SIPI	77.4	81.0	75.6	58.1	100.0	50.0
NDVI	77.4	76.2	78.0	79.0	100.0	75.0
GI	74.2	66.7	78.0	69.4	100.0	63.5
MSR	71.0	71.4	70.7	71.0	80.0	69.2
PSRI	77.4	81.0	75.6	77.4	100.0	73.1
NRI	69.4	76.2	65.9	64.5	100	57.7

**Table 6:** Comparison of the ability of PRI (570, 525, 705), ARI (860, 790, 750), and common spectral indices to discriminate between healthy wheat and wheat infected by yellow rust for data from different databases.

Index	Early-Mid Growth Stage (216 DAS, 225 DAS)			Mid-Late Growth Stage (230 DAS, 238 DAS)		
	Overall Classification Accuracy (%)	Recall		Overall Classification Accuracy (%)	Recall	
		Healthy (%)	Yellow Rust (%)		Healthy (%)	Yellow Rust (%)
PRI (700,520,575)	84.1	100	72.0	/	/	/
ARI (865,790,750)	/	/	/	93.2	100.0	90.0
PRI	81.8	100	68.0	90.9	92.9	90.0
ARI	79.5	100	64.0	90.9	100	86.7
SIPI	72.7	100	52.0	72.7	100	60.0
NDVI	77.3	100	60.0	88.6	92.9	86.7
GI	72.7	89.5	60.0	90.9	100	86.7
MSR	70.5	84.2	60.0	84.1	92.9	80.0
PSRI	72.7	100	52.0	81.8	100	73.3
NRI	70.5	89.5	56.0	90.9	100	86.7
